# A Randomized Controlled Trial of Multicomponent Exercise in Older Adults with Mild Cognitive Impairment

**DOI:** 10.1371/journal.pone.0061483

**Published:** 2013-04-09

**Authors:** Takao Suzuki, Hiroyuki Shimada, Hyuma Makizako, Takehiko Doi, Daisuke Yoshida, Kengo Ito, Hiroshi Shimokata, Yukihiko Washimi, Hidetoshi Endo, Takashi Kato

**Affiliations:** 1 Research Institute, National Center for Geriatrics and Gerontology, Obu, Aichi, Japan; 2 Section for Health Promotion, Department for Research and Development to Support Independent Life of Elderly, Center for Gerontology and Social Science, National Center for Geriatrics and Gerontology, Obu, Aichi, Japan; 3 Department of Clinical and Experimental Neuroimaging, Center for Development of Advanced Medicine for Dementia, National Center for Geriatrics and Gerontology, Obu, Aichi, Japan; 4 Department for Development of Preventive Medicine, Center for Development of Advanced Medicine for Dementia, National Center for Geriatrics and Gerontology, Obu, Aichi, Japan; 5 Department of Cognitive Disorders, Hospital of National Center for Geriatrics and Gerontology, National Center for Geriatrics and Gerontology, Obu, Aichi, Japan; 6 Department of Comprehensive Geriatric Medicine, Hospital of National Center for Geriatrics and Gerontology, National Center for Geriatrics and Gerontology, Obu, Aichi, Japan; University of Groningen, Netherlands

## Abstract

**Background:**

To examine the effect of multicomponent exercise program on memory function in older adults with mild cognitive impairment (MCI), and identify biomarkers associated with improvement of cognitive functions.

**Methodology/Principal Findings:**

Subjects were 100 older adults (mean age, 75 years) with MCI. The subjects were classified to an amnestic MCI group (n = 50) with neuroimaging measures, and other MCI group (n = 50) before the randomization. Subjects in each group were randomized to either a multicomponent exercise or an education control group using a ratio of 1∶1. The exercise group exercised for 90 min/d, 2 d/wk, 40 times for 6 months. The exercise program was conducted under multitask conditions to stimulate attention and memory. The control group attended two education classes. A repeated-measures ANOVA revealed that no group × time interactions on the cognitive tests and brain atrophy in MCI patients. A sub-analysis of amnestic MCI patients for group × time interactions revealed that the exercise group exhibited significantly better Mini-Mental State Examination (*p* = .04) and logical memory scores (*p* = .04), and reducing whole brain cortical atrophy (*p*<.05) compared to the control group. Low total cholesterol levels before the intervention were associated with an improvement of logical memory scores (*p*<.05), and a higher level of brain-derived neurotrophic factor was significantly related to improved ADAS-cog scores (*p*<.05).

**Conclusions/Significance:**

The results suggested that an exercise intervention is beneficial for improving logical memory and maintaining general cognitive function and reducing whole brain cortical atrophy in older adults with amnestic MCI. Low total cholesterol and higher brain-derived neurotrophic factor may predict improvement of cognitive functions in older adults with MCI. Further studies are required to determine the positive effects of exercise on cognitive function in older adults with MCI.

**Trial Registration:**

UMIN-CTR UMIN000003662 ctr.cgi&quest;function&hairsp;&equals;&hairsp;brows&amp;action&hairsp;&equals;&hairsp;brows&amp;type&hairsp;&equals;&hairsp;summary&amp;recptno&hairsp;&equals;&hairsp;R000004436&amp;language&hairsp;&equals;&hairsp;J.

## Introduction

Alzheimer's disease (AD) places a considerable and increasing burden on patients, caregivers and society. The number of older adults living with AD is predicted to increase from the current 26.6 million to 106.2 million by 2050 globally. [1] The current standard of care for mild to moderate AD involves treatment with acetylcholinesterase inhibitors to improve cognitive function. The *N*-methyl-d-aspartate antagonist memantine has also been reported to improve cognitive function in patients with moderate to severe AD. [2] While these drugs improve the symptoms of AD, they do not have substantial disease-modifying effects. [3] Thus, attempts have been made to identify individuals at increased risk of AD, and to test interventions that might delay the progression of prodromal symptoms of dementia.

An association has been proposed between regular participation in physical activity, especially aerobic exercise, and a variety of cognitive benefits. [4], [5], [6], [7], [8] Several meta-analyses have reported that physical activity is associated with improvements in attention, processing speed, and executive function in older adults with and without cognitive impairments. [9], [10], [11] However, these studies produced some inconsistent findings, with some reporting cognitive gains in memory function [10], [11] and other study reporting equivocal results. [9]

Evidence from neuropsychological and neuroimaging studies has suggested that mild cognitive impairment (MCI) represents a clinical prodrome to degenerative dementias such as AD. [12] For example, a population-based study in Sweden reported that the relative risks of progression to dementia in a 3-year follow-up in subjects with mild, moderate, and severe cognitive impairment (without dementia), were 3.6, 5.4, and 7.0, respectively. [13] However, of the individuals with MCI, 11% remained stable, and 25% exhibited an improvement in cognitive function between baseline and follow-up observation. [13] This variation in MCI populations should be examined to facilitate the development of interventions for inhibiting the progression of dementia. Several randomized controlled trials (RCTs) have been conducted to investigate the effects of exercise or physical activity on cognitive function in older adults with MCI. [4], [5], [6], [7], [8] These studies have revealed the effects of exercise or physical activity on cognitive function, including executive function, in older adults with MCI. However, the effect of exercise on memory function in this population remains unclear.

The precise neurobiological mechanism for the improvement of cognitive functions remains unknown, however a large number of rodent studies suggest a central role of certain molecules such as brain-derived neurotrophic factor (BDNF), insulin-like growth factor (IGF-1), and vascular endothelial growth factor (VEGF). The molecules have been shown to facilitate neurogenesis in the hippocampus, promote synaptic plasticity in the hippocampus and cerebral cortex, and angiogenesis and enhance growth and protection of neurovasculature. [14], [15] In fact, some neuroimaging studies of human subjects revealed that aerobic exercise increased hippocampal volume, [16] and gray and white matter regions including the cingulate cortex, supplementary motor cortex, inferior frontal gyrus, and superior temporal gyrus. [17]

The present randomized trial was designed to test whether a 6-month supervised multicomponent exercise program could reduce the rate of cognitive decline, especially in memory function, and reduce the rate of brain volume decline among older adults with MCI. The multicomponent exercise program included aerobic exercise, muscle strength training, and postural balance retraining, because previous reviews suggested that combined aerobic exercise and strength training interventions improved attention and working memory to a greater extent than aerobic exercise alone. [11], [18] We explored the biomarkers for identifying improvement of cognitive functions. Serum total cholesterol (T-cho), hemoglobin A1c (HbA1c), BDNF, and VEGF levels at baseline were used as potential predictors.

## Methods

CONSORT checklist and the protocol for this trial is available as supporting information; see **Checklist S1** and **Protocol S1**.

### Participants

Subjects in this study were recruited from two volunteer databases (n = 1,543), which included elderly individuals (65 years and over) selected either by random sampling or when they attended a medical check-up in Obu, Japan. Inclusion criteria specified that prospective participants were community-dwelling individuals aged 65 years and over. A total of 528 prospective participants with a Clinical Dementia Rating (CDR) of 0.5, or who complained of memory impairment, were recruited in the first round of eligibility assessments. Of these, 135 subjects satisfied the requirements of the second round of eligibility assessments, which included neuropsychological tests, which included language and memory tests, attention and executive function tests, clinical diagnosis, activities of daily living (ADL), educational level, and magnetic resonance imaging. Thirty-five subjects were excluded, meaning that a total of 100 subjects took part in the study (mean age, 75.4±7.1 years; 65–95 years, men n = 55, 51%). All subjects met the definition of MCI as per the Petersen criteria. [19] All MCI subjects had objective impairments in either episodic memory and/or executive functioning at least 1.5 standard deviations below the age-adjusted mean for at least one of the neuropsychological tests. Final classification of subjects was based on the above factors and consensus of a team of neuroscientists. Exclusion criteria included a CDR = 0, or a CDR of 1–3, a history of neurological, psychiatric, or cardiac disorders or other severe health issues, use of donepezil, impairment in basic activities of daily living (ADL), and participation in other research projects. Subjects were classified to an amnestic MCI group (aMCI) (n = 50) with neuroimaging measures, and other MCI group (n = 50) before the randomization. Then, the subjects in each group were randomly assigned to either a multicomponent exercise or an education control group using a ratio of 1∶1. Participant characteristics at the beginning of the study are shown in **Table 1**. We confirmed that there were no significant differences in demographic characteristics, physical performance, or instrumental ADL levels between the exercise and control groups. Fifty subjects with aMCI (mean age, 76.0±7.1 years; 65–92 years, men n = 27, 54%) were selected from among the subjects to participate in a sub-analysis. All subjects included the aMCI group agreed to measure functional neuroimaging tests. This sub-analysis was limited to aMCI patients because aMCI is most likely to progress to AD.[20] Objective memory impairment to determine aMCI was defined as a lower memory score on the Wechsler Memory Scale-Revised (WMS-R) Logical Memory II. [21]

**Table 1:** Characteristics of the subjects.

	All subjects		aMCI subjects	
	Exercise (n = 50)	Control (n = 50)	Exercise (n = 25)	Control (n = 25)
Age, mean (SD), y	74.8 (7.4)	75.8 (6.1)	75.3 (7.5)	76.8 (6.8)
Men, No. (%)	25 (50.0)	26 (52.0)	13 (52.0)	14 (56.0)
Educational level, mean (SD), y	10.9 (2.8)	10.4 (2.4)	11.1 (2.4)	10.8 (2.7)
Diagnosis, No. (%)				
Hypertension (3^*^, 1^†^)	23 (46.9)	22 (45.8)	13 (52.0)	11 (45.8)
Heart disease (4^*^, 1^†^)	5 (10.2)	1 (2.1)	2 (8.0)	0 (0)
Diabetes Mellitus	8 (16.0)	3 (6.0)	5 (20.0)	3 (12.0)
Medication, 3 and over (2^*^, 1^†^)	22 (44.0)	19 (39.6)	10 (40.0)	11 (45.8)
Blood pressure, mmHg				
Systolic, mean (SD)	144.6 (21.6)	142.4 (19.4)	152.2 (21.0)	143.7 (21.3)
Diastolic, mean (SD)	74.6 (11.7)	75.1 (11.2)	77.3 (11.1)	74.3 (10.1)
Blood test				
Total cholesterol, mean (SD), mg/dL	211.7 (36.2)	200.5 (34.5)	212.6 (36.9)	202.8 (32.2)
HbA1c, mean (SD), %	5.6 (0.8)	5.4 (0.5)	5.6 (0.6)	5.4 (0.5)
BDNF, mean (SD), ng/mL	12.1 (10.0)	13.5 (10.4)	11.9 (11.3)	14.4 (12.2)
VEGF, mean (SD), pg/mL	97.6 (19.7)	103.5 (22.2)	95.9 (18.4)	96.7 (15.4)
Physical performances				
Grip strength, mean (SD), kg	24.7 (8.1)	23.5 (7.3)	25.2 (7.3)	23.1 (8.4)
One legged standing, mean (SD), s	34.6 (24.6)	31.2 (23.9)	34.0 (25.1)	29.3 (23.6)
Timed up & go, mean (SD), s	8.8 (2.5)	9.2 (2.1)	9.0 (2.2)	9.1 (2.0)
IADL subscale of TMIG index, mean (SD), score	4.8 (0.9)	4.9 (0.3)	5.0 (0.2)	4.9 (0.3)
GDS, mean (SD), score	3.8 (3.1)	3.3 (2.8)	3.0 (2.1)	2.6 (2.0)
Cognitive functions, score				
MMSE, mean (SD)	26.8 (2.3)	26.3 (2.7)	26.8 (1.8)	26.6 (1.6)
ADAS-cog, mean (SD)	6.0 (2.8)	6.5 (2.8)	6.3 (2.2)	6.8 (2.2)
WMS-LM I, mean (SD)	14.6 (6.9)	13.8 (7.4)	12.5 (5.9)	12.0 (4.9)
WMS-LM II, mean (SD)	10.5 (7.4)	9.4 (7.4)	8.2 (5.4)	6.9 (5.0)
Clinical subtype, No. (%)				
Amnestic MCI	34 (68.0)	37 (74.0)		
Non-amnestic MCI	16 (32.0)	13 (26.0)		
VSRAD				
MTA-ERC atrophy, mean (SD) (1*)	1.3 (0.9)	1.5 (1.0)	1.4 (1.0)	1.4 (1.0)
WBC atrophy, mean (SD) (1*)	7.3 (4.7)	8.3 (4.6)	7.9 (3.9)	7.4 (3.3)

Abbreviations: IADL subscale of TMIG index, instrumental activities of daily living subscale of Tokyo Metropolitan Institute of Gerontology index; GDS, Geriatric Depression Scale; MMSE, Mini-Mental State Examination; ADAS-cog, Alzheimer's Disease Assessment Scale-cognitive subscale; WMS, Wechsler Memory Scale; MCI, mild cognitive impairment. ^*^missing value in all subjects. ^†^missing value in the aMCI subjects.

### Ethics

The Ethics Committee of the National Center for Geriatrics and Gerontology approved the study protocol. The purpose, nature, and potential risks of the experiments were fully explained to the subjects, and all subjects gave written, informed consent before participating in the study. The subjects had the capacity to consent because they maintained general cognitive function and daily activities.

### Interventions

The six-month, multicomponent exercise program included biweekly 90-minute sessions involving aerobic exercise, muscle strength training, postural balance retraining, and dual-task training. In addition, the exercise program included a focus on promoting exercise and behavior change. Two trained physiotherapists involved in geriatric rehabilitation conducted each intervention. Each exercise class contained 16–17 participants, and each supervised session began with a 10-min warm-up period and stretching exercise, followed by 20 min of muscle strength exercise. The subjects then practiced aerobic exercise, postural balance retraining, and dual-task training for 60 min. In the aerobic exercise and postural balance retraining, subjects underwent circuit training, including stair stepping, endurance walking, and walking on balance boards. The mean intensity of the aerobic exercise was approximately 60% of maximum heart rate which was similar to the intensity used in previous studies. [4], [6] Eleven of the 40 classes during the six-month intervention period included approximately 20–30 minutes of consecutive outdoor walking. In the dual-task training sessions, subjects performed concurrent cognitive tasks during exercise. For example, the subjects in the exercise group were asked to walk while inventing their own poem, as aerobic exercise. In the ladder training exercise, subjects learned to step in consecutive square segments, and were instructed to step as quickly and accurately as possible. Before and after each session of the program, physiotherapists conducted a health check of each subject. The physiotherapists and a well-trained instructor implemented risk management for accidents and other adverse events during the program. The subjects were instructed to carry out daily home-based muscle strength exercises and walking, which were self-monitored using a booklet and pedometer based on the concept of promoting exercise and behavior change. Attendance at each session was recorded and a transportation service was provided for participants, if necessary, to help subjects maintain their participation in the program.

Subjects in the education control group attended two education classes about health promotion during the 6-month study period. The class provided information regarding healthy diet, oral care, prevention of urinary incontinence, and health checks. However, the group did not receive specific information regarding exercise, physical activity, or cognitive health.

### Outcomes

#### Cognitive Functions

The Mini-Mental State Examination (MMSE) [22] and Alzheimer's Disease Assessment Scale-Cognitive subscale (ADAS-cog) [23] were used to assess general cognitive function.

Modified versions of the logical memory subtest from the WMS-R [21] was used to assess memory function. In the WMS-R, two short stories (Story A and B) were read aloud to the subject, who was then instructed to recall details of the stories immediately (LM I, immediate recall) and after 30 min (LM II, delayed recall; each total recall score = 50). [21]

#### MRI

MRI was performed with a 1.5-T system (Magnetom Avanto, Siemens, Germany). Three-dimensional volumetric acquisition with a T1-weighted gradient echo sequence was then used to produce a gapless series of thin sagittal sections using a magnetization preparation rapid-acquisition gradient-echo sequence (repetition time, 1700 ms; echo time, 4.0 ms; flip angle 15°, acquisition matrix 256×256, 1.3-mm slice thickness).

In analysis of brain volume, we used the voxel-based specific regional analysis system for Alzheimer's disease (VSRAD), which enables the examination of atrophy of the bilateral medial temporal areas including the entorhinal cortex (MTA-ERC) using voxel-based morphometry. [24] The VSRAD has been shown to achieve high accuracy (87.8%) in discriminating patients in the very early stages of AD with MCI from normal control subjects using Z scores. [24] A previous VSRAD study reported that atrophy of the MTA-ERC exhibited a clear functional relationship with blood flow changes in the hippocampus, thalamus and temporal lobe, which were suggested to be closely related to inter-regional anatomical and physiological connections. [25]

Acquired MRI images were formatted to gapless, transaxial images, followed by extraction of the gray matter images using SPM2. Anatomical standardization was used to fit each individual brain to standard template MRIs in the common coordinate system of the MNI T1 MRI template. [26] The segmented gray matter images were then subjected to affine and nonlinear standardization using a template of prior gray matter. The anatomically standardized gray matter images were then smoothed again using an isotropic Gaussian kernel 12 mm in full width at half maximum, to determine the partial volume effect and create a spectrum of gray matter intensities. Gray matter intensities were equivalent to the weighted average of gray matter voxels located in the volume fixed by the smoothing kernel. Regional intensity was considered equivalent to gray matter concentration. We compared the gray matter image of each patient with the mean and standard deviation (SD) of gray matter images of healthy volunteers using voxel-by-voxel Z score analysis. In the final step, the Z score was calculated according to the following equation: (Z score = ((control mean)−(individual value))/control SD). The Z score thus reflected the degree of atrophy in bilateral MTA-ERC. Higher Z scores indicated clearer MTA-ERC atrophy. VSRAD also automatically measured the degree of atrophy in the whole brain cortices (WBC), including the hippocampus: if the Z-score was more than 2.0 within a voxel, the area was considered to exhibit atrophy. [24] Thus, the proportion of atrophic area in the whole brain (%) was measured in the following way: 100×([the number of voxels with Z-score≥2.0]/[the number of whole brain voxels]).

#### Biochemical measures3

T-cho, HbA1c, BDNF, VEGF receptor 1 (VEGFR1) were used as biomarkers. Blood samples were collected between 11 am and 4 pm in a non-fasting state. The blood samples were kept at room temperature for 30 min to allow for clotting, after which the samples were centrifuged for 15 min. Serum was then harvested and stored at −25 °C until analysis. Analyses were carried out centrally in one laboratory (Special Reference Laboratories, Tokyo, Japan). BDNF and VEGFR1 were measured with the Quantikine Human kit (R&D systems, Inc. Minneapolis, MN, USA). Coefficients of variation (CVs) of BDNF in intra-assay and inter-assay precision were 2.6–3.2 and 5.5–9.8, respectively. Those of VEGFR1 were 3.8–6.2 for intra-assay and 7.6–11.3 inter-assay precision.

### Sample size

Since participants were selected on the basis of memory impairments, memory was considered the most important cognitive outcome in our study. Therefore, sample size calculations were based on AVLT data. [27] A previous study reported that a sample of 34 participants per group to detect a clinically relevant effect, with 80% power. [6] To allow for a dropout of 25%, the final sample size was 85 participants.

### Randomization–Sequence generation

Subjects were randomly assigned after completion of baseline assessments. Subjects were classified to an amnestic MCI group (n = 50) with neuroimaging measures, and other MCI group (n = 50) before the randomization. The subjects in each group were randomized to either a multicomponent exercise or an education control group using a ratio of 1∶1. The subjects were further randomized and dichotomized into two groups, an amnestic MCI group (n = 50) with neuroimaging measures, and other MCI group (n = 50).

### Randomization–Implementation and concealment

After the baseline assessment, subjects were randomized using the option “random sample of cases” in IBM SPSS statistics software (Version 19; SPSS Inc., Chicago, IL, USA). A researcher who was not aware of the aims of the study performed the randomization procedure.

### Blinding

Study personnel involved in the collection of outcome measures were blinded to the randomization assignment. Several trained speech therapists blinded to group status conducted the cognitive tests, and one speech therapist recalculated all of the results.

### Statistical methods

Statistical analysis was performed using IBM SPSS statistics software. For the baseline comparisons between exercise and control groups for all subjects, and for the amnestic MCI (aMCI) sub-analysis, Pearson's method, together with Chi square analysis with Fisher's exact test was used to investigate the categorical data. Kolmogorov-Smirnov tests confirmed that all continuous variables followed a normal distribution. Basic characteristics of patients were compared between the two groups using *t*-tests.

A general linear model for repeated-measures analysis of variance (ANOVA) was used to determine the group difference for the cognitive tests and VSRAD measurements. Two time points were treated as the within-subjects factor (effect over time) and the differences between the exercise and control groups were treated as the between-subjects factor. When the repeated-measures ANOVA indicated that the group × time interaction was significant, tests of simple main effects were performed to determine which group or groups differed significantly across the intervention period. Alpha level of the post-hoc analyses were adjusted for the Bonferroni method, i.e. corrected alpha = .025.

Multiple logistic regression models were used to determine the predictors of improvements in cognitive function. Dependent variables were the cognitive tests which showed significant improvements in the comparison between before and after the intervention of all subjects. Based on the results from the cognitive tests, the subjects were dichotomized into two categories; the subjects who improved their cognitive test scores (improvement group) and the subjects who showed no improvement, or who exhibited a deterioration in their cognitive test scores (no improvement group). Biochemical variables at baseline measurements were treated as independent variables. Covariates such as age, sex, educational level, and the intervention group were included in the logistic model.

The univariate analyses and repeated-measures ANOVA were performed with all subjects grouped together as well as with a sub-group that was limited to older adults with aMCI. The logistic regression analysis was performed to determine the predictors of improvement of cognitive functions in all subjects. All statistical significance tests were two-sided, and an alpha-level of .05 was considered statistically significant.

## Results

### Participant flow

**Figure 1:**
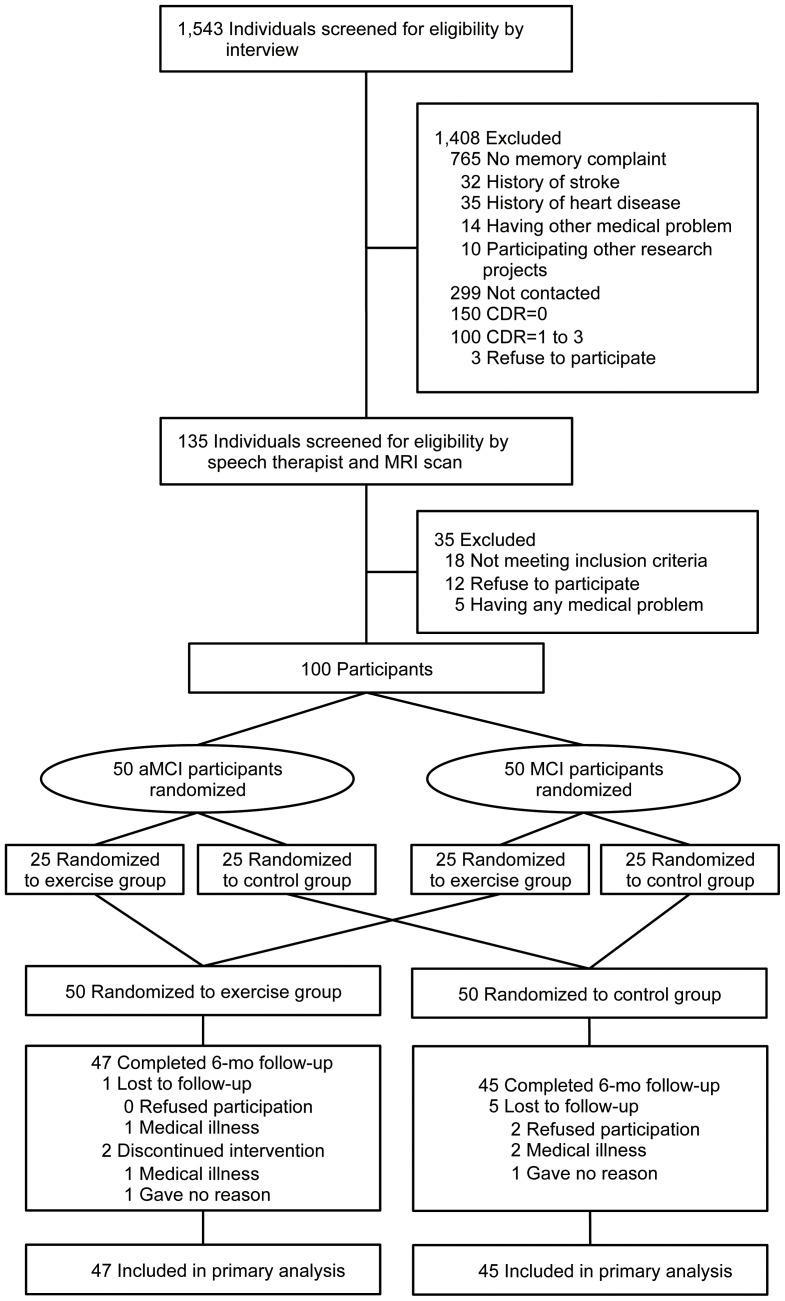
Subject flow diagram from initial contact through to study completion.

**Figure 1** shows the flow of participants from the time of screening to study completion at 6 months. Ninety-two (exercise group, n = 47) subjects completed the 6-month follow-up. Of the 50 aMCI subjects, 47 (94%) completed the 6-month follow-up. Two of the remaining 47 subjects in the exercise group (one male, one female) missed all exercise programs, but completed the examinations before and after the intervention. The two subjects were included in the following analyses. Mean adherence to the exercise program, including the remaining 47 subjects, was 85.9%, and 38 subjects (80.9%) in the exercise group attended our intervention program with greater than 80% adherence.

### Baseline data

There were no significant differences in baseline characteristics between all subjects grouped together and the aMCI group alone (**Table 1**).

### Participants analyzed

Our primary analysis of cognitive function included all patients who remained at the end of the study (total n = 92; exercise group, n = 47; control group, n = 45). A total of 90 subjects (exercise group, n = 46; control group, n = 44) completed MRI scanning. When the analyses were limited to subjects with aMCI, the exercise and control groups included 24 and 23 subjects in assessments of cognitive function and MRI, respectively.

### Outcomes in all MCI subjects

**Table 2** shows changes in cognitive scores over the 6-month period by group. There were main effects of time in ADAS-cog (*p* = .01), WMS-LM I (*p*<.01), WMS-LM II (*p*<.01), and WBC atrophy level (*p* = .03), although no main effects of group and group × time interactions were detected on the cognitive tests and brain atrophy (**Table 2**).

**Table 2:** Comparison of Cognitive Function between the Exercise and Control Group.

	All subjects (n = 100)						aMCI subjects (n = 50)					
	Mean Difference From Baseline (95% CI) in All Subjects		*P* Value ANOVA for Repeated Measures			ES	Mean Difference From Baseline (95% CI) in aMCI Group		*P* Value ANOVA for Repeated Measures			ES
	Exercise Group (n = 47)	Control Group (n = 45)	Group	Time	Group × time interaction	r	Exercise Group (n = 24)	Control Group (n = 23)	Group	Time	Group × time interaction	r
MMSE	0.2 (−0.5, 0.9)	−0.3 (−1.1, 0.4)	0.18	0.79	0.32	0.11	0.3 (−0.8, 1.3)	−1.4 (−2.5, −0.3)	0.03	0.14	.04^b^	0.31
ADAS-cog	−0.8 (−1.4, −0.2)	−0.2 (−0.8, 0.4)	0.17	0.01	.16 (1)^c^	0.15	−1.2 (−2.1, −0.3)	−0.1 (−1.0, 0.8)	0.1	0.06	0.1	0.24
WMS-LM I	2.8 (1.4, 4.2)	1.0 (−0.5, 2.4)	0.29	<.01	0.08	0.19	3.8 (1.6, 5.9)	0.5 (−1.6, 2.7)	0.14	<.01	.04^a^	0.31
WMS-LM II	3.4 (2.0, 4.8)	1.9 (0.4, 3.4)^b^	0.28	<.01	0.15	0.15	3.8 (1.8, 5.7)	2.1 (0.1, 4.2)	0.11	<.01	0.26	0.17
MTA-ERC	0 (−0, 0.1)	0 (0, 0.1)	0.18	0.08	0.89	0.02	0.1 (0, 0.2)	0 (−0.1, 0.1)	0.91	0.03	0.27	0.17
WBC	0.1 (−0.4, 0.7)	0.7 (0.1, 1.2)	0.08	0.03	0.16	0.15	−0.1 (−0.8, 0.6)	0.9 (0.2, 1.6)	0.86	0.08	<.05^b^	0.29

Abbreviations: MMSE, Mini-Mental State Examination; ADAS-cog, Alzheimer's Disease Assessment Scale-cognitive subscale; WMS, Wechsler Memory Scale; MTA-ERC, medial temporal areas including the entorhinal cortex; WBC, whole brain cortices; ES, effect size.

a *p*<.025; significant differences before versus after intervention in the exercise group

b *p*<.025; significant differences before versus after intervention in the control group

c Missing value

### Outcomes in aMCI subjects

**Figure 2:**
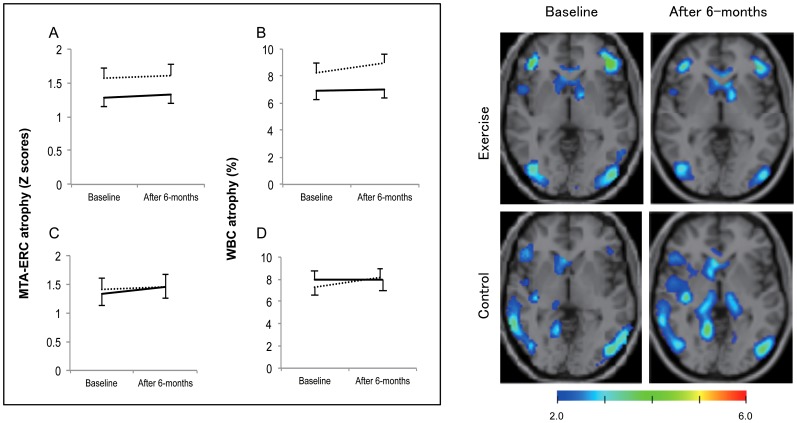
Change in MTA-ERC and WBC volumes in response to the 6-month intervention. Abbreviations: MTA-ERC, medial temporal areas including the entorhinal cortex; WBC, whole brain cortices. Left panel shows change in MTA-ERC and WBC volumes before and after the 6-month intervention. Solid and dashed lines indicate the exercise and control groups, respectively. Group mean differences and standard errors for MTA-ERC and WBC atrophy are shown in panels A and B, respectively, for all subjects. Panels C and D show mean differences and standard errors for MTA-ERC and WBC atrophy, respectively, for older adults with aMCI. The repeated-measures ANOVA revealed that there was a significant group × time interaction on WBC atrophy level (*p*<.05) in older adults with aMCI. Right panel shows typical images for VSRAD, indicated atrophy region, in subjects with aMCI in the exercise and control groups. The upper panel shows WBC atrophy in a man (81 years old) with aMCI who completed the 6-month exercise program. The rate of WBC atrophy decreased after the intervention (8.74% at baseline to 6.39% after the intervention). The lower panel shows WBC atrophy of a man (80 years old) with aMCI in the control group. The rate of WBC atrophy increased after the 6-month intervention period (7.19% at baseline to 10.48% after the intervention).

When the analyses were limited to subjects with aMCI, the repeated-measures ANOVA for MMSE showed a significant effect of group (*p* = .03) and there was a group × time interaction in MMSE (*p* = .04) indicating benefit of the exercise over time. Tests of simple main effects revealed that the control group decreased in MMSE score (*p* = .015) after intervention. A repeated-measures ANOVA showed a significant effect of time (*p*<.01) and group × time interaction (*p* = .04) in WMS-LM I. Tests of simple main effects showed that the exercise group exhibited better WMS-LM I (*p*<.01) scores compared to baseline, but not in the control group. The repeated-measures ANOVA for WMS-LM II (*p*<.01) and MTA-ERC atrophy (*p* = .03) showed a significant effect of time. However, there were no main effects of group and no group × time interactions. A repeated-measures ANOVA showed a significant group × time interaction (*p*<.05) in WBC atrophy level. There were no main effects of group or time. Tests of simple main effects revealed that the subjects in the control group showed increased WBC atrophy (*p* = .01) after intervention, compared with their baseline scores (**Table 2** **,** **Figure 2**).

### Relationships between cognitive functions and biomarkers

Paired *t*-tests revealed significant improvements in ADAS-cog (*p* = .01), WMS-LM I (*p*<.01), and WMS-LM II scores (*p*<.01) after the intervention. Multiple logistic regression analysis revealed that low T-cho level before the intervention was associated with improvement in WMS-LM I (odds ratio (OR) 0.98, 95% confidence interval (95% CI) 0.96–1.00, *p* = .02). Higher BDNF level at baseline was significantly related to improvements in ADAS-cog (OR 1.07, 95% CI 1.02–1.13, *p* = .01) independent of age, sex, educational level, and intervention (**Table 3**).

**Table 3:** Predictors of Improvements in Cognitive Function.

	ADAS-cog	*P*	WMS-LM I	*P*	WMS-LM II	*P*
	OR (95% CI)		OR (95% CI)		OR (95% CI)	
Age, years	0.97 (0.91–1.05)	.44	0.95 (0.89–1.03)	.22	0.96 (0.90–1.04)	.34
Sex, women/men	1.00 (0.35–2.82)	1.00	0.74 (0.26–2.13)	.57	2.56 (0.85–7.66)	.09
Educational level, years	0.85 (0.70–1.04)	.11	0.93 (0.76–1.13)	.45	1.01 (0.83–1.22)	.96
Intervention, exercise group/control group	2.85 (1.10–7.37)	.03	2.27 (.90–5.72)	.08	1.98 (.77–5.12)	.16
T-cho, mg/dl	1.00 (0.98–1.02)	.96	**0.98 (0.96–1.00)**	**.02**	0.99 (0.97–1.01)	.18
HbA1c, %	0.53 (0.25–1.14)	.10	1.20 (0.57–2.53)	.64	0.61 (0.29–1.30)	.20
BDNF, ng/ml	**1.07 (1.02–1.13)**	**.01**	1.00 (0.95–1.05)	.94	1.02 (0.97–1.08)	.39
VEGFR1, pg/ml	0.99 (0.97–1.01)	.39	0.99 (0.96–1.01)	.32	1.00 (0.98–1.03)	.74

Abbreviations: OR, odds ratio; 95% CI, 95% confidence interval; ADAS-cog, Alzheimer's Disease Assessment Scale-cognitive subscale; WMS, Wechsler Memory Scale; T cho, total cholesterol; HbA1c, hemoglobin A1c; BDNF, brain-derived neurotrophic factor (BDNF); VEGFR1, vascular endothelial growth factor receptor 1.

Missing values: ADAS-cog (n = 10), WMS-LM I (n = 9), WMS-LM II (n = 9)

### Adverse events

Four subjects (exercise group, n = 2; control group, n = 2) experienced adverse events (hospitalization for illness). Falls (as a type of minor adverse event) over a 6-month period were reported by 23/90 (26%) of subjects, with no significant differences among groups. There were no other adverse events during exercise intervention for 6-months.

## Discussion

### Evidence of exercise on cognitive function

Older adults with MCI have been found to exhibit greater decreases in memory function than in other cognitive functions, relative to healthy older adults. [28] The enhancement of cognitive function, especially memory function, in individuals with MCI may play a crucial role in preventing the progression from MCI to AD in older adults. Klusmann et al. reported significant effects of a multifaceted exercise program on cognitive function, finding that a 6-month exercise program resulted in improvements in delayed story recall. [29] However, their sample consisted of healthy, well-functioning females without any signs of cognitive impairment. In addition, previous studies reported that aerobic exercise or other physical activity can increase executive function in older adults with cognitive impairments, but the effects of exercise on memory function in this population remain unclear. [4], [5], [6], [7], [8] To our knowledge, this is the first study to demonstrate an improvement in logical memory following multicomponent exercise training among older adults with aMCI. The exercise group showed significant differences not only in WMS-LM I scores, but also in MMSE scores compared to the control group in aMCI populations. Our intervention study extends the results of previous studies with healthy samples, indicating the potential for an increase in memory performance and maintenance of general cognitive function in subjects exhibiting signs of cognitive decline.

A meta-analysis of aerobic exercise and neurocognitive performance demonstrated that interventions combining aerobic exercise and strength training, similar to our program, improved attention, processing speed and working memory to a greater extent than aerobic exercise alone. [11] However, the mechanism underlying this improvement remains unclear. A previous study reported that subjects with MCI improved their episodic memory performance when they were exposed to a multifactorial cognitive intervention program that included dual-task attentional and memory training. [30] Dual-task deficit is recognized as a potential early marker for dementia, [31], [32] and dual-task-related changes in performance were greater in subjects with MCI compared with cognitively normal age-matched controls. [33], [34] Our multicomponent program involved changes in cognitive load using dual-task stimulation and learning tasks. We believe that dual-task training may have a greater effect on various cognitive functions, for example, general and memory functions, than interventions that only focus on aerobic exercise. [7], [10] However, the results from the present study do not provide direct evidence for the positive effect of dual-task training. Future studies are required to investigate the effects of dual-task training on cognitive function in the older adults with MCI.

Lautenschlager et al. reported that physical activity and behavioral intervention improved general cognition among adults with MCI. [4] The multicomponent exercise training in the current study also included encouragement for subjects to engage in more physical activity. Our results further support the notion that training involving physical activity can have a beneficial effect not only on memory function, but also on general cognitive function in people with aMCI. General cognitive function can be used to discriminate between people who progress to AD and those who do not. [35] Improvements of memory function and maintenance of general cognitive function suggest that multicomponent exercise can help prevent progression from MCI to AD. However, despite significant interactions, the effect sizes in general cognitive function and logical memory were small. Moreover, these interactions would not become significant if the p-values were adjusted for multiple comparisons. Further studies are required to determine the positive effects of exercise on cognitive function in older adults with MCI.

### Relationship between exercise and brain atrophy

It is well established that structures in the medial temporal lobe, particularly the hippocampus and ERC, are essential for normal memory function. There is an emerging literature describing baseline structural MRI correlates of cognitive impairment in elderly adults with mild cognitive impairment (MCI) and Alzheimer's disease (AD). Some studies have identified relationships between aerobic exercise and increased brain volume [16], [17] and functional connectivity between parts of the frontal, posterior, and temporal cortices [36] in healthy older adults. For example, Erickson et al. found that the hippocampus remains plastic in late adulthood and that a 1-year period of aerobic exercise was sufficient for enhancing volume. [16] Our 6-month multicomponent exercise program with MCI subjects revealed that exercise did not have a significant group × time interaction on MTA-ERC scores or WBC atrophy compared to the control group. However, there was significant group × time interaction in WBC atrophy level, when tested in a sub-analysis restricted to aMCI subjects. Post-hoc analyses revealed that the control group exhibited increased WBC atrophy after intervention, compared with their baseline scores. These results suggest that older adults with aMCI may exhibit high levels of plasticity in WBC atrophy. Further study is needed to establish our findings using large samples and detailed neuroimaging analysis.

### Predictors of increasing of cognitive function

In the relationships between cognitive function and biochemical measures, low T-cho and high BDNF serum levels at baseline were associated with increased memory and general cognitive function in the MCI subjects, respectively. Serum lipoprotein levels may be a common and potentially modifiable risk factor for AD. [37] For example, a prospective study reported that lower serum levels of LDL and T-cho were associated with better cognitive performance and a lower risk of cognitive impairment in 1,037 women with cardiovascular disease. [38] Our finding extends knowledge about the relationships between T-cho and cognitive function to older adults with MCI. Animal studies have revealed that the structure and function of the hippocampus, a brain region critical for certain forms of cognition, is adversely affected by hyperlipidemia. (e.g. [39]) Abnormal lipid metabolism may be undesirable status for improvement cognitive functions, especially memory. Exercise is also a valid and feasible way to manage lipoprotein levels and regular activity may be potential strategies for preventing cognitive decline in elderly individuals. [40]

One of the main determinants of cell size is cell growth, which is modulated by certain growth factors, such as BDNF. The levels of BDNF-associated gene expression have been found to increase with physical activity. [14] BDNF expression has also been suggested to play a role in learning and synaptic plasticity. [41] The present results indicate that high serum BDNF levels have a beneficial effect on general cognitive function in older adults with MCI.

### Limitations

The present study involved several limitations. The small sample size should be addressed by replication with a larger group of adults with MCI. Of the 135 potential subjects screened for eligibility in our study, 35 were excluded for not meeting inclusion criteria, refusal to participate, or medical reasons (**Figure 1**). This selection bias may have affected the generalizability of our findings to population-based samples. Other limitations include unknown group differences in risk factors of cognitive decline and AD, such as apolipoprotein E ε4 genotype, and inflammation, although there were no significant differences between groups in terms of hypertension, diabetes mellitus, medications, biomarkers of lipid metabolism, physical performance, instrumental ADL functioning, or depressive mood. In addition, it is possible that the improvement in the exercise group resulted from the social contact to which the intervention group was exposed. This possibility cannot be completely excluded with the present design, and should be addressed in future studies.

### Conclusion

The current results indicate that a multicomponent exercise program can provide cognitive benefits for older adults with aMCI. The effects of exercise were most pronounced for logical memory and general cognitive function in older adults with aMCI. Exercise was found to maintain the atrophy levels of the whole brain cortex in older adults with aMCI. Improvement of cognitive function was associated with low T-cho and high BDNF levels at baseline. A future follow-up investigation is required to determine whether the observed effects are associated with prevention or delayed onset of AD in older adults with MCI.

## Supporting Information

Checklist S1 **CONSORT Checklist.**
(DOC)

Protocol S1 **Trial Protocol.**
(DOCX)
