# Supplementary material for: A Randomized Controlled Trial of Multicomponent Exercise in Older Adults with Mild Cognitive Impairment
Source: PLoS One. 2013 Apr 9;8(4):e61483. doi: 10.1371/journal.pone.0061483 (PMC3621765; doi:10.1371/journal.pone.0061483)
Supplement: Protocol S1 — Trial Protocol. (DOCX) [file pone.0061483.s002.docx]

**Protocol S1**

A randomized control trial for preventing decline of cognitive functions

in the community living elderly with mild cognitive impairment

Protocol


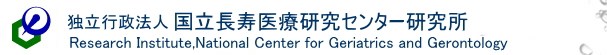
National Center for Geriatrics and Gerontology

Obu, Aichi, Japan

**March, 2010**

# 1. Purpose of the Study

Dementia, which increases with age, disrupts the life of both the patient himself and his family and requires substantial treatment and elderly nursing costs, so establishing methods to prevent and treat it has become an urgent task. Especially when considering the rapidly changing demographics of Japan, the problems involved in dementia are becoming an increasingly important issue. Currently, no radical treatment has been established for Alzheimer's disease and cerebrovascular disease, which are the primary causative diseases of dementia, and so initiatives for preventing dementia or delaying its onset play an important role as countermeasures to dementia.

A state that is not dementia but rather a mild cognitive decline is called "mild cognitive impairment" (or "MCI"), which has attracted attention as a preliminary stage for preventing dementia. A large-scale epidemiological study focusing on community-dwelling elderly in Japan put the prevalence of MCI at roughly 5% to 7%. It was reported that 3.7% of these community-dwelling elderly people with MCI transitioned to dementia within three years, whereas 0.2% of elderly people who did not have MCI developed dementia. Also, because 38.5% of cases of MCI return to normal after five years [[1](#_ENREF_1)], initiatives to mitigate MCI are important in order to prevent dementia.

Approaches to mitigate MCI and prevent the onset of dementia have made use of drug therapy, the purpose of which is to eliminate risk factors and delay onset, as well as non-drug therapy, which has included improvements in lifestyle habits. Examples of drug therapies include administering drugs for hypertension, hyperlipidemia, and diabetes, which are risk factors for Alzheimer's disease and cerebrovascular disease, or prescribing donepezil hydrochloride in order to delay the onset of Alzheimer's-type dementia. However, the direct effects of drug therapy to eliminate risk factors are difficult to grasp, and donepezil hydrochloride can be expected to have only a limited effect [[2](#_ENREF_2)].

Examples of non-drug therapies used to intervene in order to prevent dementia include promoting habitual physical activity [[3](#_ENREF_3),[4](#_ENREF_4)], eating foods high in antioxidants and/or anti-inflammatory ingredients [[5](#_ENREF_5)], and participation in social and intellectual activities [[6](#_ENREF_6)], which have been recognized as protective factors against the onset of dementia. In particular, many findings have been noted regarding a connection between aerobic exercise and preventing the onset of Alzheimer's disease, and the results of a recent randomized control trial have been reported as providing verification of the efficacy of exercise for elderly adults with MCI [[7](#_ENREF_7)]. In that study, 152 participants were randomly assigned to an aerobic exercise group and a non-aerobic exercise group, and to a vitamin B group and a placebo group, and a one-year intervention was carried out. The participants exercised twice weekly, for 60 minutes each time. For aerobic exercise, they trained walking together in groups. The results showed that aerobic exercise has no significant effect in improving cognitive function. However, these results were based on an intention-to-treat analysis, which included 30 participants who did not attend the exercise sessions. If only those elderly adults who had a high attendance rate among the aerobic exercise group are included in the analysis, then the results show increased memory and attentiveness, confirming the effectiveness of aerobic exercise in elder adults with MCI, though only to a limited extent. In another recent report, when elderly adults with MCI (a mean age of 70 years) participated in aerobic exercise four times every week over the course of six months with a heart rate reserve of 75% to 85%, the results showed that executive function was significantly improved [[8](#_ENREF_8)].

Exercise thus serves as a potential mechanism with a favorable effect on cognitive function, and, findings from animal studies especially have suggested that it reduces nerve inflammation, promotes angiogenesis, helps regulate the neuroendocrine response, and reduces amyloid accumulation. A recent study on humans has also reported that six months of aerobic exercise increased the capacity in the regions of the brain associated with lowered cognitive function caused by aging [[9](#_ENREF_9)]. This is thought to be due to the fact that aerobic exercise leads to angiogenesis and increases the flow of blood to the brain [[10](#_ENREF_10)].

Given the background of these studies, it is an object of the present study to examine whether it is possible for exercise to also improve cognitive function in elderly adults with MCI. The present study further seeks to use a randomized controlled trial to verify whether improved brain capacity and brain function are observed in tandem with functional changes.

# 2. Participants

This research project seeks to validate the effect of exercise intervention in order to validate the effect of improved cognitive function in elderly adults with MCI. Elderly adults over the age of 65 who speak Japanese as their native language will be randomly assigned to either a control group or an exercise-intervention group. Participants who are found to have amnestic MCI will be subjected to randomized, stratified allocation in which they are assigned into either an Exercise Group 1 of people who agree to have their brain function measured by positron emission tomography (PET) testing or near infrared spectroscopy (NIRS), or into an Exercise Group 1 of the other elderly adults with MCI.

The reason why it is necessary to randomly allocate the participants is that the effect of intervention cannot be validated if the influence on cognitive function of unknown factors other than intervention is not equalized among the groups, and high-quality scientific evidence cannot be obtained without this technique.

In terms of ethical issues for the participants in the random allocation of the participants, due consideration must be given in general in the practice of health services for the elderly to ensure that all residents have equal access to appropriate services. The elderly adults with MCI who will be considered as candidate participants in this research project are a population that, having been diagnosed with MCI, requires some form of action. However, because there currently is a lack of any sufficiently clear and effective method for mitigating MCI with non-drug intervention, we find ourselves in a situation where it is not possible to decide what appropriate services would be. Therefore, at this point in time, randomly allocating the participants has the potential to incur advantages and disadvantages in each group; if this comes to be better understood and there is thus a desire to conduct a research project, we feel that ethical considerations can be avoided. However, it is necessary to proceed with the present research project under the assumption that, in the event that the effects of intervention are confirmed, a similar intervention will immediately be provided to the control group. The effects of intervention must be judged on the comprehensive observation of trends in evaluation indices conducted six months and 12 months after intervention is begun.

## 2-1. Participant Inclusion Criteria

The criteria for participant inclusion will be that participants must be elderly adults over the age of 65 who had a clinical dementia rating (CDR) of 0.5 in an epidemiological study conducted in Ōbu City (contributing researcher: Hiroshi Shimokata) with the aid of the Ministry of Health's 2009 Grant-in-Aid for Scientific Research (anti-dementia general research projects) or who received a specific health examination in Ōbu City in 2009 and have had problems with subjective memory. Participants will sign a written agreement; participants who are deemed incapable of giving consent will be allowed to participate under the condition that a family member or relative agree to sign as legal representative.

The participants included in Exercise Group 1 are patients with amnestic MCI. The criteria for amnestic MCI follows the criteria of the Japan-Alzheimer’s disease neuroimaging initiative (J-ADNI) shown below.

Criteria for Amnestic MCI

**Type I**: The patient complains of own memory impairment + People in the position of caregivers (family) accept this

**Type II**: The patient does not complain of own memory impairment, but rather the existence of the memory impairment is indicated by the caregivers (family).

Note: This excludes cases in which only the patient complains of memory impairment and people in the position of caregivers (family) do not accept this.

MMSE is from 24 to 30 points.

The cut-off scores with the Wechsler memory Scale-R/Logical memory II are as follows, considering how many years of education the patient has had.

0-9 years of education: 2 points or less

10-15 years of education: 4 points or less

16 or more years of education: 7 points or less

CDR is 0.5. The patient must not suffer from depression.

## 2-2. Participant Exclusion Criteria

1. Patients with Parkinson's disease, multiple cerebral infarction, Huntington's disease, normal pressure hydrocephalus, brain tumor, progressive supranuclear palsy, epilepsy, subdural hematoma, multiple sclerosis, head trauma with lingering delayed effects, or diabetes.

2. Patients found by MRI at the time of screening to have an infection, or a localized lesion such as cerebral infarction that will affect cognitive function.

3. Patients who have received a pacemaker, an aneurysm clip, a prosthetic valve, a cochlear implant, or any other kind of metal in the body.

4. Patients who have major depression, bipolar disorder, a history of alcoholism or other kind of drug-dependence, or severe or unstable disease.

5. Patients who have a vitamin B12 or folic acid deficiency, syphilis, or abnormal thyroid function.

6. Patients who are participating in a drug trial for a novel AD drug treatment.

7. Patients under the age of 85 who are found to have significant functional decline in motor function testing and for whom performing the exercise intervention is deemed to be a challenge.

## 2-3. Allocation of Participants

Participants will go through three rounds of testing for 1) CDR score and subjective memory impairment, 2) cognitive function and motor function, and 3) MRI photography, following which they will be randomly allocated to a control group and an exercise group. Prior to this allocation, elderly adults with amnestic MCI will be categorized to perform a stratified allocation. The method for allocating participants involves having experts, who knew neither the study content nor the participants, randomly assign participants into equally sized groups using a computer program, which they will do while blinded.

# 3. The Survey

The following table lists the sequence before and after intervention in which, for each patient, blood is sampled, cognitive function is tested, motor function is tested, vital function is examined, the brain is CAT-scanned, and - in some adults - brain activity is measured by NIRS and a PET scan is taken.

**Study Measurement Schedule**

３—１．Exercise Group 1

| Items | Screening | BL | 6M | 12M |
| --- | --- | --- | --- | --- |
| Explain study & obtain consent | X | X |  |  |
| Screening of inclusion and exclusion criteria | X | X |  |  |
| Medical interview | X | X | X | X |
| Vital sign |  | X | X | X |
| MMSE | X | X | X | X |
| CDR | X | X | X | X |
| Wechsler memory Scale-R/  Logical Memory |  | X | X | X |
| Digit span |  | X | X | X |
| Stroop color word test |  | X | X | X |
| Digit symbol substitution |  | X | X | X |
| Category Fluency |  | X | X | X |
| Trail making test A and B |  | X | X | X |
| Geriatric Depression Scale |  | X | X | X |
| Clock Drawing |  | X | X | X |
| ADAS-Cog |  | X | X | X |
| Physical performance |  | X | X | X |
| TMIG index |  | X | X | X |
| Basic ADL |  | X | X | X |
| Life-space assessment |  | X | X | X |
| SF-8 |  | X | X | X |
| MRI（1.5T） |  | X | X | X |
| NIRS |  | X | X | X |
| FDG-PET |  | X | X | X |
| Blood sampling |  | X | X | X |
| Payment for participants with neuroimaging tests |  | X | X | X |
| Check harmful event |  | X | X | X |
| Check self-monitoring exercise |  |  | X | X |

３—２．Exercise Group 2

| Items | Screening | BL | 6M | 12M |
| --- | --- | --- | --- | --- |
| Explain study & obtain consent | X | X |  |  |
| Screening of inclusion and exclusion criteria | X | X |  |  |
| Medical interview | X | X | X | X |
| Vital sign |  | X | X | X |
| MMSE | X | X | X | X |
| CDR | X | X | X | X |
| Wechsler memory Scale-R/  Logical Memory |  | X | X | X |
| Digit span |  | X | X | X |
| Stroop color word test |  | X | X | X |
| Digit symbol substitution |  | X | X | X |
| Category Fluency |  | X | X | X |
| Trail making test A and B |  | X | X | X |
| Geriatric Depression Scale |  | X | X | X |
| Clock Drawing |  | X | X | X |
| ADAS-Cog |  | X | X | X |
| Physical performance |  | X | X | X |
| TMIG index |  | X | X | X |
| Basic ADL |  | X | X | X |
| Life-space assessment |  | X | X | X |
| SF-8 |  | X | X | X |
| MRI（1.5T） |  | X | X | X |
| NIRS |  | X | X | X |
| FDG-PET |  | X | X | X |
| Blood sampling |  | X | X | X |
| Payment for participants with neuroimaging tests |  | X | X | X |
| Check harmful event |  | X | X | X |
| Check self-monitoring exercise |  |  | X | X |

３—３．Control Group

| Items | Screening | BL | 6M | 12M |
| --- | --- | --- | --- | --- |
| Explain study & obtain consent | X | X |  |  |
| Screening of inclusion and exclusion criteria | X | X |  |  |
| Medical interview | X | X | X | X |
| Vital sign |  | X | X | X |
| MMSE | X | X | X | X |
| CDR | X | X | X | X |
| Wechsler memory Scale-R/  Logical Memory |  | X | X | X |
| Digit span |  | X | X | X |
| Stroop color word test |  | X | X | X |
| Digit symbol substitution |  | X | X | X |
| Category Fluency |  | X | X | X |
| Trail making test A and B |  | X | X | X |
| Geriatric Depression Scale |  | X | X | X |
| Clock Drawing |  | X | X | X |
| ADAS-Cog |  | X | X | X |
| Physical performance |  | X | X | X |
| TMIG index |  | X | X | X |
| Basic ADL |  | X | X | X |
| Life-space assessment |  | X | X | X |
| SF-8 |  | X | X | X |
| MRI（1.5T） |  | X | X | X |
| NIRS |  | X | X | X |
| FDG-PET |  | X | X | X |
| Blood sampling |  | X | X | X |
| Payment for participants with neuroimaging tests |  | X | X | X |
| Check harmful event |  | X | X | X |

# 4. The Intervention

## 4-1. Exercise Intervention

Duration: 90 minute exercise classes, twice weekly, over a period of six months, and six months of follow-up (a total of 12 months)

Location: In an indoor space greater than 60m^2^, and outdoors in the vicinity of the meeting place

Content: The exercise content focuses on aerobic exercise and is conducted in conjunction with strength training as well, in order to improve the basic ability to operate. The table below shows an example of how the classes will be operated. The exercise will be conducted in small groups of 20 people or fewer, in order to consider the safety of the elderly participants and to give the groups a sense of togetherness. The intervention will be run by six or more people - the people responsible for leading the exercise (physiotherapists) and supporters (volunteer staff). The physiotherapists observe the participants' states and emphasize individual support to ensure an appropriate exercise load, and the volunteer staff watches to provide safety control in consideration of all participants. The number of intervention dropouts is thought to be dramatically reduced when the sense of belonging to a group is high, and so to that end, the same staff members will preferably continue the intervention. Before every class there will be a check for vitals like blood pressure; exercise begins once the participants' physical condition can be confirmed. Also, each class begins with a warm-up and ends with a cool-down, which focuses on stretching, in careful consideration to allow the participants to exercise safely. The participants will also be provided with an at-home exercise menu, to encourage them to increase the amount of activity on days on which there is no class, as well as with a self-report, by which their exercise activity can be checked.

Contents of Intervention

| **Group** | **Number of subjects** | **Program** | **Time** | **Stuffs** |
| --- | --- | --- | --- | --- |
| **Control** | n = 50 | Educational lecture | ３hours | Two lecturer |
| **Intervention** | n = 50 | Exercise  90 min/time, 2 times/week, >6 months | 10:00－11:30  13:00－15:00  15:30－17:00 | Physical therapist: 2~4  Assistant: 4 |

### 4-1-1. Intervention staff

Two to four physiotherapists and three to four volunteer staff

### 4-1-2. Aerobic exercise

Duration: about 30 minutes

The aerobic exercise is designed to focus on walking and stair training. The aerobic exercise will be performed over about 20 to 30 minutes per class. Referring to previous studies [[8](#_ENREF_8),[11](#_ENREF_11)] that carried out an aerobic exercise intervention for elderly adults with MCI, the objective is to start from a heart rate reserve load of 40% to 50% and gradually increase intensity over the course of six weeks to reach an exercise intensity of 75% to 85% heart rate reserve after week 7 (Table). The exercise intensity is set by the physiotherapists, who will use the Karvonen method [[12](#_ENREF_12)] to set the exercise intensity based on heart rate reserve, and also will refer to the rated perceived exertion (or "RPE") of the participants to adjust the exercise intensity in consideration of their safety. The participants' pulses will also be monitored and managed during exercise.

The content of the aerobic exercise draws on ordinary indoor and outdoor walking as well as step training (using a bar or ladder). It will also adopt an aerobic exercise (double challenge) program incorporating a cognition challenge which, when combined with the exercise, is expected to activate brain activity.

In the special case of adults who are taking a β-blocker, their medications status will be checked in advance, and their exercise intensity will be studied on an individual basis rather than being set based on heart rate.

### 4-1-3. Strength training

Duration: about 20 minutes

The exercise intervention will involve strength training using resistance exercise aimed at improving the basic ability to operate. The targeted muscles will be groups of large muscles, especially the muscles around the shoulder joints, the muscles of the trunk, and the muscles around the hip joints, the knee joints, and the ankle joints. To prevent pain or injury, the four-week period at the start of the intervention will be low-intensity strength training aimed at invigorating muscle activity and promoting becoming more active. From week 5 onward, the intensity will gradually increase, using, for example, weight bands where the intensity can be set by adjusting the weight, or using Thera-bands, which have an adjustable intensity. The physiotherapists will be responsible for managing setting the loads and adjusting them by consulting the participants' RPE, while remaining alert for the occurrence of any pain or injury. The 1RM (repetition maximum) of each muscle will be measured as needed to study how to set the loads.

Time schedule of exercise

| Time | Tasks |
| --- | --- |
| 10：00～10：10  （10 min） | Check of health condition and implementation of home exercise |
| 10：10～10：40  （30 min） | Streching, and muscle strength exercise as home-based exercise |
| 10：40～11：50  （10 min） | Rest; water replacement |
| 11：50～11：20  （30 min） | Aerobic exercise |
| 11：20～11：30  （10 min） | Cool-down; stretching |

## 4-2. Control Group

The control group will be advised to change their lifestyle as little as possible during the intervention period. Forethought will also be given to prevent the disadvantage to the control group from being obvious, by having them attend briefings on the measurement results and conducting health lectures.

## 4.3. Terminating the intervention

The intervention must terminate and the evaluation results must be collected within 12 months during the 2010 fiscal year, but from the standpoint of preventing dementia, it would be preferable to have an initiative that continues on for a longer time. The content of the intervention is being altered to be more inexpensive to continue conducting a sustainable intervention. The plan is to set the intervention frequency to at least once a week and continue the exercise classes with the volunteer staff.

# 5. Method for describing the study to the participants and obtaining their consent

The participants will be given informed consent forms approved by the ethical review boards of each institution and receive a thorough explanation, both written and oral. Voluntary written agreements will then be obtained from the participants.

Whenever any information regarding efficacy and/or safety deemed to affect the consent of the participants is obtained, or whenever the execution plan is altered in any way that would affect the consent of the participants, this information will be promptly provided to the participants. The participants' re-consent will then be obtained by first confirming in advance that they agree to participate in the study, and then obtaining the approval of the ethical review boards in advance in order to modify the informed consent forms.

# 6. Standards and methods for disclosing data to the participants

## 6-1. Brain imaging

Of the brain images taken, template responses (for example, "the entire brain has mild atrophy") regarding the primary findings from each MRI and FDG-PET that is evaluated and measured will be drafted to be given to desiring participants. However, these are limited to explaining and commenting on the results; the images will not be used to provide any disease diagnoses (for example, "The image indicates Alzheimer's disease"). In the event that any findings should be incidentally discovered, including brain tumors, vascular malformation, subdural hematoma, lacunar infarct, or advanced lesions in the periventricular white matter, care will be taken to disclose this to the participant and provide them with support.

## 6.2. Performance tests and blood tests

The results from general tests will explained to everyone, as a general rule, and utilized as a preventative measure against lifestyle diseases.

# 7. Criteria for Discontinuation

1. Patients who decline to participate in the study or who withdraw their consent.

2. Patients who are found after registration not to fulfill the requirements for eligibility.

3. Patients who struggle to continue the study due to progressive complications.

4.Patients who struggle to continue due to an adverse event.

5. In the event that the study is discontinued altogether, when it is deemed appropriate to discontinue the study for other reasons.

6. Patients who discontinue due to an adverse event receive long-term follow-up as much as possible during the study period.

# 8. Treatment for adverse events

## 8-1. Responding to participants when an adverse event occurs

When an adverse event takes place, the people conducting the research will take appropriate action immediately and, whenever necessary, quickly provide transport to a medical facility. A record of this will be taken without contradiction in a case report form. Whenever treatment for an adverse event becomes necessary, this will be conveyed to the participants.

## 8-2. Reporting of Serious Adverse Events

All serious adverse events that occur during the study period and all serious adverse events that occur after the termination (discontinuation) of the study and are suspected of having a connection to the study will be reported. When an adverse event is confirmed to have occurred, a report will immediately be provided to a supervisor and head researcher.

# 9. Consideration for the participants' rights, safety, and disadvantages

Utmost consideration will be given to protecting the participants' confidentiality when documenting raw data and handling consent forms pertaining to the study. The results of the study, when published, will not contain any information that could be used to identify the participants. It will be mentioned that the data on the participants obtained during the study shall not be used for any purpose other than the objectives of the study. The participants' privacy will be kept confidential and consideration will be given to ensure that absolutely no personal information be leaked. The research representative is responsible for managing the data. Anonymized data will be disclosed, but individual data will not be published.

# 10. Cost Burden on the Participants

The participants shall bear none of the costs incurred by the tests or interventions required in this study. However, tests or drugs required to treat the primary disease and complicating diseases shall not be borne by the present study.

# 11. Compensation for health problems and obtaining insurance

## 11-1. Compensation for health problems

No compensation will be offered for losses caused by any lawful act.

## 11.2 Obtaining liability insurance

To prepare for any liability, the research staff will be insured against liability. Recreational insurance will be obtained for participation in the exercise intervention classes.

# 12. Complying with the Declaration of Helsinki

This study will be conducted in compliance with the Declaration of Helsinki.

# 13. Archiving

Essential documents relating to the conduct of the study are kept in the National Center for Geriatrics and Gerontology. The principal researcher is responsible for their storage. The documents will be kept for a minimum of five years after the termination of the study period. Tables corresponding to the participants' identities will be kept separately and anonymized. Documents will be disposed of by shredder following the end of the storage period.

# 14. Publication of research results

The anonymized data will be put through a statistical analysis and published sequentially. The analysis results will be disclosed in the form of papers, conference presentations, reports, and mass media publications.

# 15. Research Funds and Conflicts of Interest

The present study will be conducted using commissioned project funds from Ōbu City of Aichi Prefecture. The planning, implementation, and reporting of the present study will be free of any "potential conflicts of interest" that would affect the results of the study or the interpretation of the results, and the rights and interests of the participants will not be neglected in the implementation of the study.

# 16. Changes to the Implementation Plan etc.

In the event that there are any changes to the implementation plan or the informed consent form, the advanced approval of the ethical review committees will be required.

# Appendix 1

## Physical performance tests

**Date：　　/ /**

ID ：

**Name：**

【Blood pressure】

①　SBP（　　　　）　DPB（　　　　）　HR（　　　）

②　SBP（　　　　）　DPB（　　　　）　HR（　　　）

【Body composition】

Height　 （　　　　．　）cm

Weight　 （　　　　．　）kg

% body fat（　　　　．　）%

【Grip strength】

（　　　　　）kg　　( right / left )

【Knee extension strength】

①（　　　　　）N

②（　　　　　）N　　Arm（　　　　．　）cm　　( right / left )

【One-legged standing time】

①（　　　　．　）s　　　②（　　　　．　）s

【5mwalking speed】

Comfortable　　（　　　　．　）s　　Steps　（　　　　　）

Maximum

①（　　　　．　）s　　Steps　（　　　　　）

②（　　　　．　）s　　Steps　（　　　　　）

【5mwalking speed with cognitive-task】

Comfortable　　（　　　　．　）s　　Steps　（　　　　　）

【5mwalking speed with motor-task】

Comfortable　　（　　　　．　）s　　Steps　（　　　　　）

【Timed Up & Go】

Comfortable　　（　　　　．　）s

Maximum

①（　　　　．　）s

②（　　　　．　）s

【6 min walk test】

Distance（　　　　　　　）m　　　　RPE（　　　　　）

【Reaction time】

Simple RT

①（　　　　）ms

②（　　　　）ms

③（　　　　）ms

④（　　　　）ms

⑤（　　　　）ms

Prove RT (cognitive task)

①（　　　　）ms

②（　　　　）ms

③（　　　　）ms

④（　　　　）ms

⑤（　　　　）ms

Prove RT (motor task)

①（　　　　）ms

②（　　　　）ms

③（　　　　）ms

④（　　　　）ms

⑤（　　　　）ms

## Questionnaire

**Date：　　/ /**

ID ：

**Name：**

Anamnesis

Current medical history

【Medication】

| Antihypertensive | （ □No □Yes ） |
| --- | --- |
| Stabilization agent | （ □No □Yes ） |
| Sleep agent | （ □No □Yes ） |
| Cardiac medication | （ □No □Yes ） |
| Cerebral medication (circulation or metabolism) | （ □No □Yes ） |
| Diabetes or hyperlipidemia drags | （ □No □Yes ） |
| Antidepressant agent | （ □No □Yes ） |
| Antipsychotic agent | （ □No □Yes ） |
| Ginkgo biloba | （ □No □Yes ） |
| Others |  |

Dominant hand

Right / Left

Drug allergy

No / Yes（　　　　　　　　　　　　　　　　　　　　　　）

Medical history

| Any operation | （ □No □Yes ） |
| --- | --- |
| Cardiac pacemaker or prosthetic valve | （ □No □Yes ） |
| Artificial ear | （ □No □Yes ） |
| Metal fragment in the body | （ □No □Yes ） |
| Blood vessel prosthesis | （ □No □Yes ） |
| Operation of cerebral aneurysm | （ □No □Yes ） |
| Parkinson’s disease | （ □No □Yes ） |
| Multiple infarction | （ □No □Yes ） |
| Huntington's disease | （ □No □Yes ） |
| Normal pressure hydrocephalus | （ □No □Yes ） |
| Brain cancer | （ □No □Yes ） |
| Progressive supranuclear palsy | （ □No □Yes ） |
| Epilepsia | （ □No □Yes ） |
| Subdural hematoma | （ □No □Yes ） |
| Multiple sclerosis | （ □No □Yes ） |
| Head trauma | （ □No □Yes ） |
| Other brain disease | （ □No □Yes ） |
| Diabetes | （ □No □Yes ） |
| Depression or bipolar disorder | （ □No □Yes ） |
| Alcohol or drug dependence | （ □No □Yes ） |
| Deficiency of VB12 or folic acid | （ □No □Yes ） |
| Lues venerea | （ □No □Yes ） |
| Thyroid dysfunction |  |
| Medication for Alzheimer’s disease |  |

Inhibition of exercise from a primary care doctor

No / Yes（reason: ）

## Mini- Mental State Examination (MMSE)

Name of Subject: _____________________________ Age: ______________________

Name of Examiner: ____________________________ Date of Examination: _________

Approach the patient with respect and encouragement

- Ask: Do you have any trouble with your memory? Yes No
- May I ask you some question about your memory? Yes No

**SCORE ITEM**

**5 ( ) Time Orientation**

Ask:

- What is the year? **_________________ (1),**
- Season? **________________________ (1),**
- Month of the year? **________________ (1),**
- Date? **__________________________ (1),**
- Day of the week? **__________________ (1).**

**5 ( ) Place Orientation**

Ask:

- Where are we now? What is the state? **___________________(1),**
- City? **______________________________________________(1),**
- Part of the city? **______________________________________(1),**
- Building? **___________________________________________(1),**
- Floor of the building? **_________________________________(1).**

**3( ) Registration of Three Words**

Say: Listen carefully. I am going to say three words. You say them back after I stop. Ready? Here they are… PONY (wait 1 second) QUARTER (wait 1 second) ORANGE (wait 1 second) what are those words?

- **_________________________ (1)**
- **_________________________ (1)**
- **_________________________ (1)**

Give 1 point for each correct answer, and then repeat them until the patient learns all three.

**5( ) Serial 7’s as a Test of Attention and Calculation**

Ask: Subtract 7 from 100 and continue to subtract 7 for each subsequent remainder until I tell you to stop.

- What is 100 take a way 7? **___________________________(1)**

Say: Keep going

- **_________________________________(1)**
- **_________________________________(1)**
- **_________________________________(1)**
- **_________________________________(1)**

**3( ) Recall of Three Words**

Ask:

What were those three words I asked you to remember?

- **_________________________________(1)**
- **_________________________________(1)**
- **_________________________________(1)**

**2( ) Naming**

Ask:

What is this? (Show pencil) **__________________ (1)**
 what is this? (Show watch) **__________________** **(1)**

**1( ) Repetition**

Say:

Now I am going to ask you to repeat what I say. Ready? ***NO IFS AND OR BUTS***

Now you say that **____________________________________ (1)**

**3( ) Comprehension**

Say:

Listen carefully because I am going to ask you to do something.

Take this paper at your left hand **(1),** fold it in half **(1),** and put it on the floor **(1)**

**1( ) Reading**

Say:

Please read the following and do what it says, but do not say it aloud. **(1)**

***CLOSE YOUR EYES***

**1( ) Writing**

Say:

Please write a sentence. If patient does not respond say: write about the weather. **(1)**

**1( ) Drawing**

Say: Please copy this design

**TOTAL SCORE ______________** Assess level of consciousness along a continuum

**________________________________________**

**Alert Drowsy Stupor Coma**

## Wechsler memory Scale-R/Logical memory II

Immediate　　　　　　points,　Delay　　　　　　　points

## Digit span

3・8　　　forward　　　　　, backward

4・1・5　　　forward　　　　　, backward

3・0・8・7　　　forward　　　　　, backward

8・5・2・9・6　　　forward　　　　　, backward

8・1・1・3・0・1　　　forward　　　　　, backward

1・9・9・7・4・6・0　　　forward　　　　　, backward

4・6・5・7・8・8・3・9　　　forward　　　　　, backward

## Stroop color word test

①Neutral　 Time（　　　　　）, 　Responses（　　　　　）,　　Incorrect answers（　　　　）

②Congruent Time（　　　　　）, 　Responses（　　　　　）,　　Incorrect answers（　　　　）③Incongruent Time（　　　　　）, 　Responses（　　　　　）,　　Incorrect answers（　　　　）

## Word Fluency test

Category

Animal （　　　）

Fruits （　　　）

Vehicle （　　　）

Letter

“shi” （　　　）

“i” （　　　）

“re” （　　　）

## Trail making test (TMT), part A, B

Trail making test, part A

Last number（　　　　　）　　　Time（　　　　　）s

Trail making test, part B

Last number or letter（　　　　　）　　　Time（　　　　　）s

## GERIATRIC DEPRESSION SCALE (SHORT VERSION)

| **No:** | **Questions:** | **Answer:** |
| --- | --- | --- |
| 1. | Are you basically satisfied with your life? | Yes / No |
| 2. | Have you dropped many of your activities or interests? | Yes / No |
| 3. | Do you feel that your life is empty? | Yes / No |
| 4. | Do you often get bored? | Yes / No |
| 5. | Are you in good spirits most of the time? | Yes / No |
| 6. | Are you afraid that something bad is going to happen to you? | Yes / No |
| 7. | Do you feel happy most of the time? | Yes / No |
| 8. | Do you feel helpless? | Yes / No |
| 9. | Do you prefer to stay at home, rather than go out and do things? | Yes / No |
| 10. | Do you feel that you have more problems with memory than most? | Yes / No |
| 11. | Do you think it is wonderful to be alive now? | Yes / No |
| 12. | Do you feel pretty worthless the way you are now? | Yes / No |
| 13. | Do you feel full of energy? | Yes / No |
| 14. | Do you feel that your situation is hopeless? | Yes / No |
| 15. | Do you think that most people are better off then you are? | Yes / No |
|  | **Total Score** |  |

## TMIG Index

| a. | Can you use public transportation by yourself? | Yes / No |
| --- | --- | --- |
| b. | Are you able to shop for daily necessities? | Yes / No |
| c. | Are you able to prepare meals by yourself? | Yes / No |
| d. | Are you able to pay bills? | Yes / No |
| e. | Can you handle your own banking? | Yes / No |
| f. | Are you able to fill out forms for your pension? | Yes / No |
| g. | Do you read newspapers? | Yes / No |
| h. | Do you read books or magazines? | Yes / No |
| i. | Are you interested in news stories or programs dealing with health? | Yes / No |
| j. | Do you visit the homes of friends? | Yes / No |
| k. | Are you sometimes called on for advice? | Yes / No |
| l. | Are you able to visit sick friends? | Yes / No |
| m. | Do you sometimes initiate conversations with young people? | Yes / No |

## Basic ADL

| a. | Can you take a meal by yourself? | Yes / No |
| --- | --- | --- |
| h. | Can you change of clothes by yourself? | Yes / No |
| l. | Can you walk by yourself? | Yes / No |
| m. | Can you take a bath by yourself? | Yes / No |

## Life-space assessmet

# Appendix 2

## Booklet of home-based exercise

## Self-monitoring sheet

# Memory tasks during exercise

## Stick step training

・

## Ladder steep training

## Circuit training

| 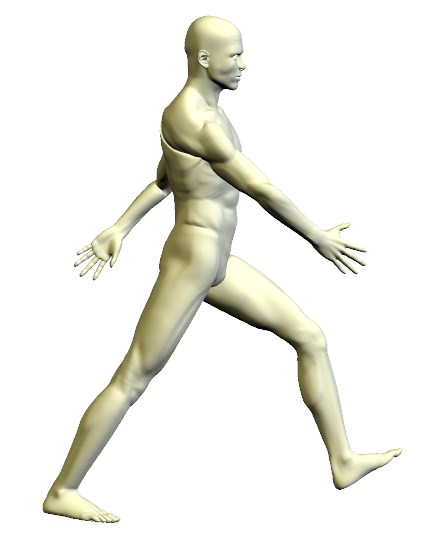 | [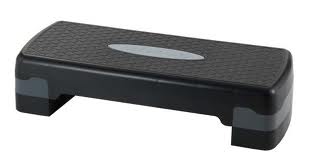](http://www.google.co.jp/imgres?imgurl=http://ecx.images-amazon.com/images/I/31eoUEmOUkL.jpg&imgrefurl=http://diet.goo.ne.jp/guide/sports/15322711/B003TJ9C6C/?ranking=37&usg=__umJdJE8Mu4_l6bvSmfKSniQTQzY=&h=265&w=500&sz=15&hl=ja&start=96&sig2=fHiijNR_P0ZHT4-zywfmXg&zoom=1&tbnid=O2XEIquX-lmviM:&tbnh=89&tbnw=168&ei=iMSFTNDLLYy8vQOg6c3IBA&prev=/images?q=%E3%82%B9%E3%83%86%E3%83%83%E3%83%97%E5%8F%B0&um=1&hl=ja&sa=N&biw=1436&bih=732&tbs=isch:1&um=1&itbs=1&iact=hc&vpx=259&vpy=433&dur=140&hovh=163&hovw=309&tx=155&ty=90&oei=ZsSFTMD1MpSmvQPa-PGSBA&esq=8&page=4&ndsp=31&ved=1t:429,r:1,s:96) |
| --- | --- |
| Walk | Box step |
|  |  |
|  |  |
| Stick step | Ladder step |


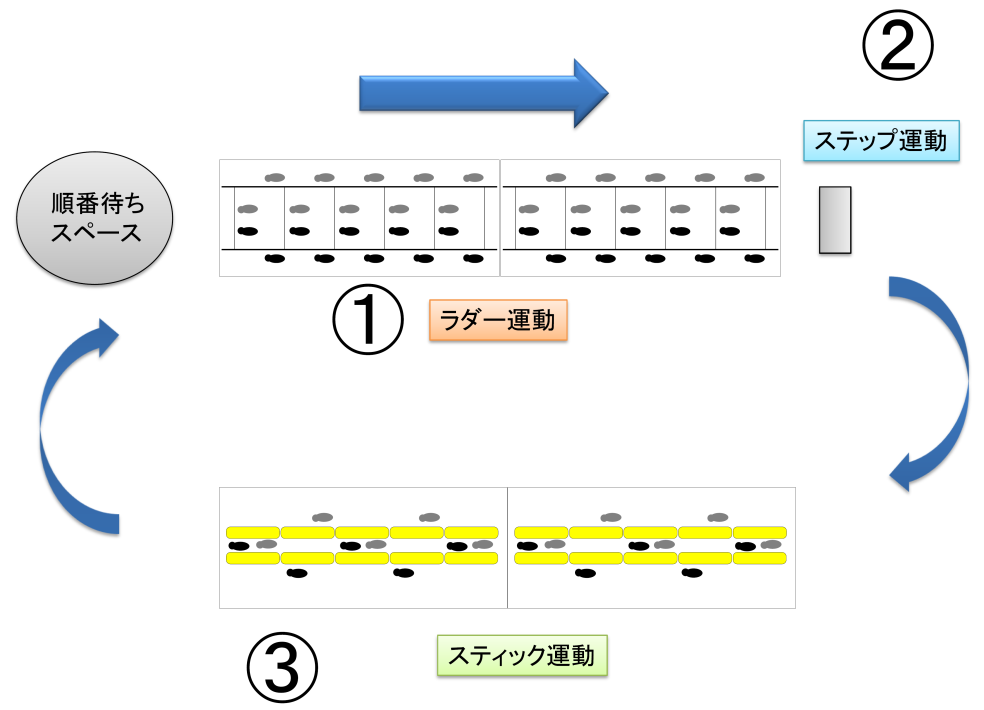


# １９．References

1. Ishikawa T, Ikeda M, Matsumoto N, Shigenobu K, Brayne C, et al. (2006) A longitudinal study regarding conversion from mild memory impairment to dementia in a Japanese community. Int J Geriatr Psychiatry 21: 134-139.

2. Petersen RC, Thomas RG, Grundman M, Bennett D, Doody R, et al. (2005) Vitamin E and donepezil for the treatment of mild cognitive impairment. N Engl J Med 352: 2379-2388.

3. McIntosh GA (2011) Comments on the affordable care act and the future of clinical medicine. Annals of internal medicine 154: 141; discussion 143-144; author reply 144.

4. Galindo CL, McIver LJ, Tae H, McCormick JF, Skinner MA, et al. (2011) Sporadic breast cancer patients' germline DNA exhibit an AT-rich microsatellite signature. Genes, chromosomes & cancer.

5. van Zyl GU, van Mens TE, McIlleron H, Zeier M, Nachega JB, et al. (2011) Low lopinavir plasma or hair concentrations explain second line protease inhibitor failures in a resource-limited setting. Journal of acquired immune deficiency syndromes.

6. Lord S, Chastin SF, McInnes L, Little L, Briggs P, et al. (2011) Exploring patterns of daily physical and sedentary behaviour in community-dwelling older adults. Age and ageing 40: 205-210.

7. Kim SJ, Nian C, McIntosh CH (2011) Adipocyte expression of the glucose-dependent insulinotropic polypeptide receptor (GIPR) involves gene regulation by PPAR{gamma} and histone acetylation. Journal of lipid research.

8. Baker LD, Frank LL, Foster-Schubert K, Green PS, Wilkinson CW, et al. (2010) Effects of aerobic exercise on mild cognitive impairment: a controlled trial. Arch Neurol 67: 71-79.

9. Cohen SA, McIlvried DE (2011) Impact of computer-assisted data collection, evaluation and management on the cancer genetic counselor's time providing patient care. Familial cancer.

10. Crossan GP, van der Weyden L, Rosado IV, Langevin F, Gaillard PH, et al. (2011) Disruption of mouse Slx4, a regulator of structure-specific nucleases, phenocopies Fanconi anemia. Nature genetics 43: 147-152.

11. Colcombe SJ, Erickson KI, Scalf PE, Kim JS, Prakash R, et al. (2006) Aerobic exercise training increases brain volume in aging humans. J Gerontol A Biol Sci Med Sci 61: 1166-1170.

12. Karvonen MJ, Kentala E, Mustala O (1957) The effects of training on heart rate; a longitudinal study. Ann Med Exp Biol Fenn 35: 307-315.
